# Subaortic pannus causing complete outlet obstruction after bioprosthetic aortic valve replacement in a patient with left ventricular assist device: a case report

**DOI:** 10.1093/ehjcr/ytae592

**Published:** 2024-11-05

**Authors:** Rohan Joshua Krishnaswamy, Vanathi Sivasubramaniam, Desiree Robson, Christopher Simon Hayward, Kavitha Muthiah

**Affiliations:** Heart and Lung Transplant Unit, St Vincent’s Hospital, Darlinghurst, Sydney, NSW 2010, Australia; Faculty of Medicine, University of New South Wales, Kensington, NSW 2033, Australia; Heart and Lung Transplant Unit, St Vincent’s Hospital, Darlinghurst, Sydney, NSW 2010, Australia; Faculty of Medicine, University of New South Wales, Kensington, NSW 2033, Australia; Department of Anatomical Pathology, St Vincent’s Hospital, Darlinghurst, Sydney, NSW 2010, Australia; Heart and Lung Transplant Unit, St Vincent’s Hospital, Darlinghurst, Sydney, NSW 2010, Australia; Heart and Lung Transplant Unit, St Vincent’s Hospital, Darlinghurst, Sydney, NSW 2010, Australia; Faculty of Medicine, University of New South Wales, Kensington, NSW 2033, Australia; Victor Chang Cardiac Research Institute, Darlinghurst, Sydney, NSW 2010, Australia; Heart and Lung Transplant Unit, St Vincent’s Hospital, Darlinghurst, Sydney, NSW 2010, Australia; Faculty of Medicine, University of New South Wales, Kensington, NSW 2033, Australia; Victor Chang Cardiac Research Institute, Darlinghurst, Sydney, NSW 2010, Australia

**Keywords:** Continuous-flow LVADs, Subaortic pannus, Bioprosthetic aortic valve replacement, Left ventricular outflow tract obstruction, Case report

## Abstract

**Background:**

Subaortic pannus formation complicates bioprosthetic aortic valve (AV) replacement. We report an extreme case in a continuous-flow left ventricular assist device (LVAD) patient.

**Case summary:**

A 49-year-old Caucasian female with dilated cardiomyopathy was bridged to transplant with a HeartWare Ventricular Assist Device (Medtronic). Duration of support was prolonged, 6 years & 7 months, due to allosensitization requiring desensitization. Pump thrombosis occurred 2 years & 4 months post-LVAD requiring alteplase thrombolysis. The patient underwent bioprosthetic AV replacement 3 years & 10 months post-LVAD for symptomatic aortic incompetence. Transthoracic echocardiography (TTE) performed 1 year and 2 years post-bioprosthetic AV replacement repeatedly demonstrated an AV closed during all cardiac cycles without incompetence and nil flow through the left ventricular outflow tract (LVOT). Following transplant, analysis of explanted heart revealed a fused AV. A pannus adherent to the underside of the AV had formed across the entire AV outlet, with complete obliteration of LVOT. This subaortic pannus was not visualized on previous TTE. Histologically, the pannus consisted of hypocellular fibrous tissue with chronic inflammatory cells, spindle histiocytes, and myofibroblasts scattered throughout the loose fibromyxoid stroma, the latter highlighted on CD68 immunohistochemical stain (IHC). Partial endothelialization on the pannus surface was highlighted on ERG and CD31 IHC. Neither calcification nor signs of acute inflammation were noted. In contrast to previous cases, there was no evidence of associated thrombus macroscopically or microscopically.

**Discussion:**

Prolonged LVAD support may facilitate subaortic pannus following bioprosthetic AV replacement due to AV closure and altered transvalvular flow. Due to the parallel LVAD circulation, subaortic pannus may develop asymptomatically, without haemodynamic compromise, allowing progression to total LVOT obstruction. This requires consideration prior to LVAD explantation in bridge-to-recovery patients.

**Learning points**

- Bioprosthetic aortic valves are subject to a unique and complex interaction during left ventricular assist device (LVAD) support.
- Continuous-flow LVAD support may facilitate the formation of subaortic pannus in patients with a coexisting bioprosthetic aortic valve replacement. This can result in left ventricular outflow tract obstruction. The formation of subaortic pannus may be clinically asymptomatic due to the parallel LVAD circulation.
- In LVAD patients with bioprosthetic aortic valves, subaortic pannus formation can confound myocardial recovery assessment and prevent device weaning.

## Introduction

Increasing numbers of continuous-flow left ventricular assist device (cfLVAD) patients are requiring bioprosthetic aortic valve (AV) replacements to correct aortic incompetence (AI) induced by the deleterious haemodynamic effects of cfLVAD support. While durable, these bioprostheses are susceptible to degeneration, which is accelerated during cfLVAD support.^1,2^ Hence, understanding the interplay and risks of prolonged cfLVAD support with coexisting bioprosthetic AV replacement is essential for clinicians who manage these complex patients. We report a case with this cfLVAD–bioprosthesis interaction that resulted in an extreme occurrence of subaortic pannus causing complete left ventricular outflow tract (LVOT) obstruction.

## Summary figure

**Table:** 

Timeline of events	
9 January 2016	LVAD inserted as bridge to transplant.
	Aetiology of heart failure: dilated cardiomyopathy.
24 July 2016	Driveline infection requiring 6 weeks of IV benzylpenicillin, then indefinite oral amoxicillin prophylaxis.
9 May 2018	Pump thrombosis of the inflow cannula confirmed on transthoracic echocardiography. This necessitated ICU admission and alteplase thrombolysis.
15 October 2019	TTE performed following symptomatic dyspnoea and high LVAD flows. Findings: LVEDD: 53 mm, LVEF: 15–20%. AV: moderate continuous AI, AV remains closed throughout the cardiac cycle. RV: moderately dilated with moderate-to-severe dysfunction. Tricuspid valve: severe incompetence with hepatic vein systolic flow reversal.
4 November 2019	Bioprosthetic AV replacement and concomitant tricuspid valve ring annuloplasty. Patient transferred to ICU post-surgery.
10 November 2019	Day-6 post-operative TTE findings:
	LVEF: 15–20%. Bioprosthetic AV: nil AI, AV likely remains closed throughout the cardiac cycle as there is no forward flow on Doppler interrogation through the LVOT. Tricuspid valve: mild-to-moderate incompetence.
28 October 2020	Progress TTE:
	LVEDD: 58 mm, LVEF: 15–20%. Bioprosthetic AV: nil AI, AV likely remains closed throughout the cardiac cycle as there is no forward flow on Doppler interrogation through the LVOT and AV. Tricuspid valve: nil residual incompetence.
10 November 2021	Progress TTE:
	LVEDD: 61 mm, LVEF: 15–20%. Bioprosthetic AV: nil AI, AV remains closed throughout the cardiac cycle.
15 June 2022	Progress TTE:
	LVEDD: 55 mm, LVEF: 10–15%. Bioprosthetic AV: poorly visualized, nil AI.
7 August 2022	Orthotopic heart transplantation; subaortic pannus identified during LVAD explantation.

AV, aortic valve; ICU, intensive care unit; IV, intravenous; LVAD, left ventricular assist device; LVEDD, left ventricular end-diastolic diameter; LVEF, left ventricle ejection fraction; RV, right ventricle; TTE, transthoracic echocardiography.

## Case report

We describe a 49-year-old Caucasian female who underwent emergent implantation with a HeartWare Ventricular Assist Device (HVAD; Medtronic, Minneapolis, MN) in January 2016 as a bridge to transplant. The aetiology of heart failure was dilated cardiomyopathy, diagnosed during the peripartum period at age 19. Despite initial stabilization, she progressively deteriorated from age 33 onwards. Otherwise, her past medical history included paroxysmal atrial fibrillation (diagnosed 2012), a dual-chamber implantable cardioverter defibrillator (implanted August 2015), stage 3a chronic kidney disease (baseline estimated glomerular filtration rate: 45 mL/min/1.73 m^2^; reference range: ≥60), and a thyroidectomy for thyrotoxicosis (2015). Initial physical examination performed upon admission found her to be in respiratory distress with accessory muscle use and respiratory rate 30/min, hypotensive (76/42 mmHg), and in atrial fibrillation with a rapid ventricular response (130 b.p.m.). However, oxygen saturation was 97% on room air. The jugular venous pressure was not elevated, heart sounds were dual with no murmur, no lung crepitations were heard, and only mild pedal oedema was palpable.

The duration of LVAD support before heart transplantation was prolonged, 6 years & 7 months, due to sensitization with strong human leucocyte antigen class I and II antibodies and calculated panel reactive antibodies (cPRAs) > 99.9% due to prior pregnancies. This required hospital admission for desensitization protocols in April 2019, November 2021, and July 2022. Anticoagulation was achieved with aspirin and warfarin, with an international normalized ratio (INR) target of 2–3.

Driveline infection occurred in July 2016 (6 months post-LVAD implant), following complaints of nausea and abdominal pain. On ultrasound, a collection at the driveline was visualized, identified via swabs as *Streptococcus dysgalactiae*. Growth was also identified in urine cultures, suggesting bacteraemia. A 6-week course of intravenous benzylpenicillin was completed, and the patient was started on long-term oral amoxicillin prophylaxis until device explant.

Pump thrombus occurred in May 2018 (2 years & 4 months post-LVAD). Suspicion was raised by complaints of anorexia, nausea, dyspnoea, and jaundice during routine follow-up. Investigations revealed an elevated lactate dehydrogenase (1950 U/L; reference range: 120–250) and subtherapeutic INR (1.8; target: 2–3). Transthoracic echocardiography revealed a mobile echodensity at the opening of the inflow cannula, consistent with thrombus. Thrombolysis was achieved with alteplase, and the target INR was subsequently uptitrated to 2.7–3.

**Figure 1:**
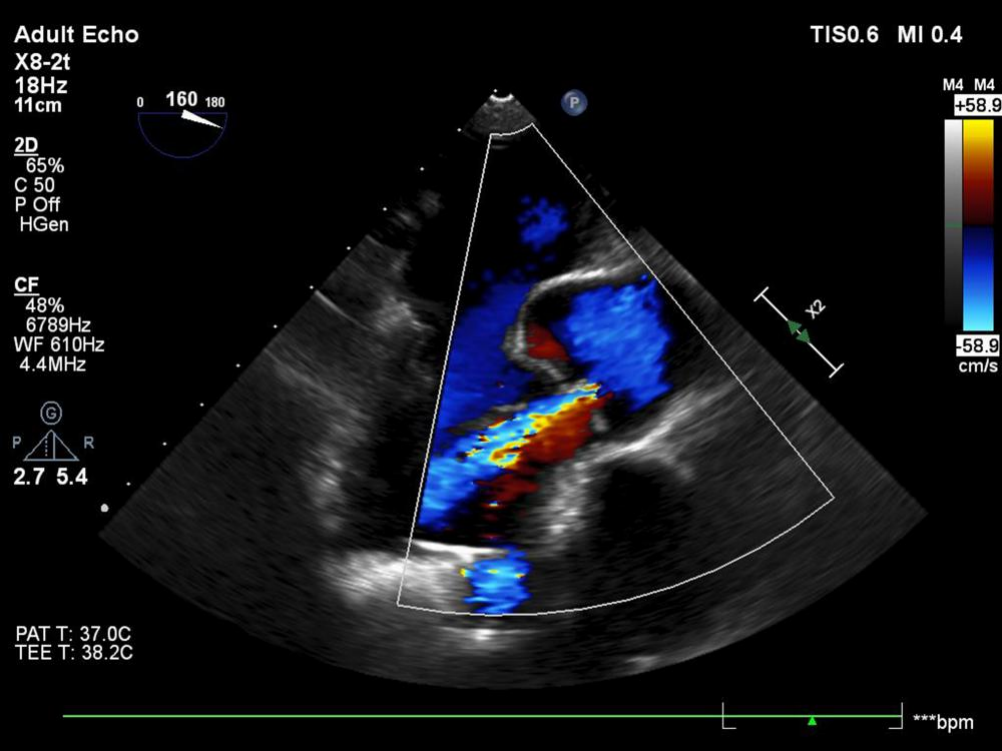
Mid-oesophageal long-axis view on transoesophageal echocardiography, October 2019, demonstrating aortic valve closure and moderate continuous aortic incompetence.

**Figure 2:**
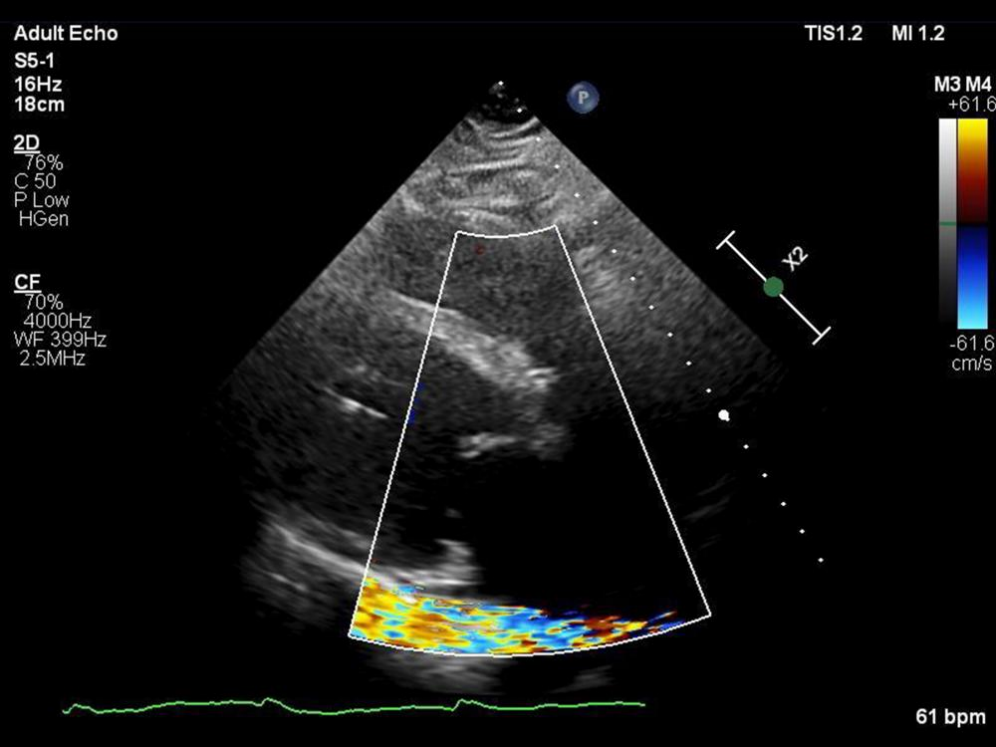
Parasternal long-axis view on transthoracic echocardiography, June 2022, demonstrating resolution of aortic incompetence 2 years and 7 months post-bioprosthetic aortic valve replacement.

Aortic incompetence was diagnosed in October 2019 following dyspnoea and high LVAD flows. Transthoracic and transoesophageal echocardiography revealed a closed AV throughout the cardiac cycle with moderate continuous AI, predominantly arising from the left coronary cusp commissures (*Figure 1* and Supplementary material online, *Video S1*). Moderate-to-severe right ventricular dysfunction and severe tricuspid incompetence were also identified. In November 2019 (3 years & 10 months post-LVAD), the patient underwent a bioprosthetic AV replacement (25 mm Edwards Perimount Magna Ease) and concomitant tricuspid valve repair (30 mm Edwards Physio Tricuspid annuloplasty band). The post-operative course was complicated by right ventricular dysfunction that required continuation of milrinone until post-operative day 8. Day 6 post-operative TTE demonstrated a satisfactorily functioning bioprosthetic AV, which remained closed throughout the cardiac cycle with no incompetence and a structurally normal tricuspid valve with mild-to-moderate incompetence. Routine TTE surveillance performed to monitor valvular incompetence at 1 year, 2 years, and 2 years & 7 months (*Figure 2* and Supplementary material online, *Video S2*) post-bioprosthetic AV replacement repeatedly demonstrated a closed AV with no incompetence.

**Figure 3:**
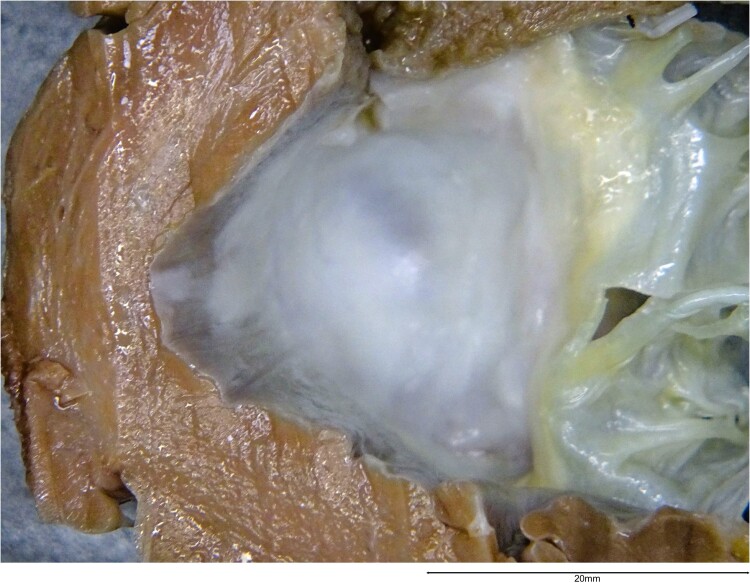
Macroscopic view of the subaortic pannus from the left ventricular side demonstrating a pale, smooth, and dome-shaped fibrous region completely obstructing the aortic outflow tract.

**Figure 4:**
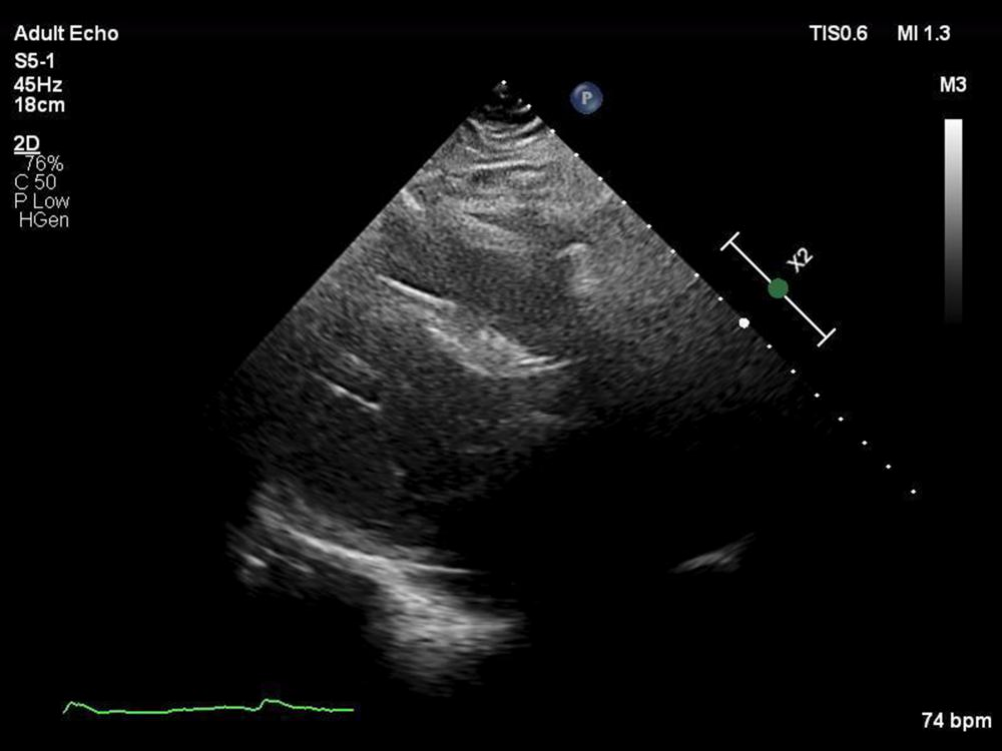
The subaortic pannus was not discernible on a parasternal long-axis view on transthoracic echocardiography, June 2022.

Device explant and heart transplant occurred in August 2022 (age 55) following perioperative intravenous immunoglobulin, plasma exchange, and eculizumab. Pathological analysis of the explanted heart revealed a fused bioprosthetic AV. There was a smooth, pale, dome-shaped area of pannus on the ventricular side of the bioprosthetic AV (*Figure 3*) which, upon sectioning, extended and adhered to the underside of the valve. The subaortic pannus extended across the entire AV outlet, causing complete obliteration of the LVOT. This subaortic pannus was not discernible on previous TTE (*Figure 4* and Supplementary material online, *Video S3*).

**Figure 5:**
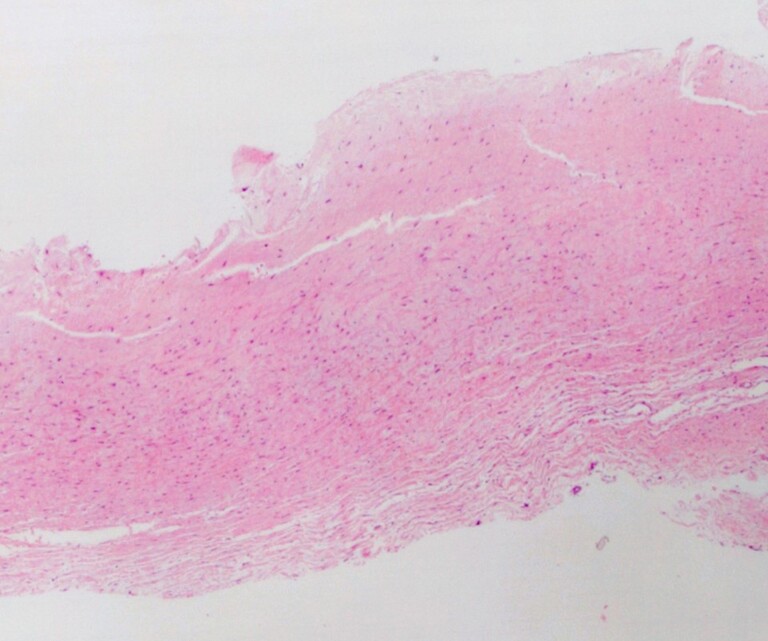
Section through the subaortic pannus with haematoxylin and eosin stain at ×40 magnification. Sections show hypocellular fibrous tissue with chronic inflammatory cells, spindle histiocytes, and myofibroblasts scattered throughout the loose fibromyxoid stroma. Partial endothelization is noted on the surface of the pannus.

Microscopically, sections through the subaortic pannus exhibited classical pannus formation of hypocellular fibrous tissue (*Figure 5*). This extended from the insertion point of the bioprosthetic AV (areas of suture material and foreign body giant cell reaction) across to the opposite surface of valve insertion. Chronic inflammatory cells, spindle histiocytes, and myofibroblasts were scattered throughout the loose fibrocollagenous tissue, the latter highlighted via CD68 immunohistochemical stain (IHC). Partial endothelization was noted on the surface of the pannus, highlighted on ERG and CD31 IHC. Scattered smooth muscle formation within the pannus was highlighted via SMA IHC. There were no appreciable signs of calcification or acute inflammation. Lastly, there was no evidence of associated thrombus macroscopically or microscopically.

## Discussion

We have demonstrated an extraordinary case of complete LVOT obstruction due to subaortic pannus formation found at explant in a patient with prolonged cfLVAD support and coexisting bioprosthetic AV, and have included pathological analysis. As more cfLVAD-supported patients undergo bioprosthetic AV replacement, it is essential to understand their long-term interactions to guide management decisions.^3^

We propose that prolonged cfLVAD support may facilitate subaortic pannus formation due to sustained functional AV closure and altered transvalvular flow.^2,4^ Although asymptomatic during normal pump functioning, in instances of cfLVAD failure (e.g. mechanical dysfunction or occlusive thrombus) whereby flow through the AV is essential to sustain cardiac output, the development of subaortic pannus would result in sudden death.^5–7^

Secondly, subaortic pannus formation can confound assessment of myocardial recovery in bridge-to-recovery patients and prevent cfLVAD weaning in an otherwise recovered heart.^2,5,6^ To make this more difficult, the subaortic pannus was clinically undetectable, further preventing clinicians from identifying these instances whereby pannus formation is confounding assessment. Due to the parallel LVAD circulation, subaortic pannus may develop asymptomatically, without haemodynamic compromise, allowing silent progression to total LVOT obstruction. Meanwhile, the inability to visualize the subaortic pannus on TTE could be secondary to poor acoustic windows in LVAD patients. This implores caution when weaning support in cfLVAD patients with bioprosthetic AVs, especially those with prolonged support duration and persistent AV closure, which may portend pannus formation.^2^ Patel *et al*.^8^ have suggested gated retrospective cardiac 4D computed tomography scans to screen for bioprosthetic AV fusion pre-cfLVAD weaning. Studies should evaluate whether this would assist with subaortic pannus visualization. Research is also needed to create protocols to guide cfLVAD weaning in affected patients, including guidelines regarding surgical correction of the subaortic pannus, should the need arise intraoperatively. Lastly, in patients who achieve recovery and cfLVAD explant despite the subaortic pannus, persistence of the pannus may serve as a nidus for microthrombi and portend systemic embolism and stroke.^9^

Case reports exist describing subaortic LVOT obstruction due to thrombus in LVAD patients with bioprosthetic AVs, reflecting subaortic blood stasis and haemo-incompatibility.^5,6,10^ In contrast, our case exhibited no features of concomitant thrombus on the pannus surface. While our patient underwent thrombolysis, this occurred prior to bioprosthetic AV replacement. No signs of a subaortic thrombus or pannus were visualized intraoperatively during the AV replacement.

The subaortic pannus was comprised of myofibroblasts and chronic inflammatory cells, suggesting it was the result of a reactive, chronic inflammatory process. Therefore, we speculate that the pannus formation is the result of an excessive foreign body giant cell inflammatory reaction to the bioprostheses that has been able to progress to complete LVOT obstruction in the context of prolonged cfLVAD support and functional AV closure.^11,12^ While we do not believe that the desensitization protocols themselves directly augmented pannus formation, the prolonged support duration in our case was a result of the patient’s significant sensitization status. Hence, we conjecture that this phenomenon would not occur in cfLVAD patients with native AVs. Indeed, no pannus was visualized intraoperatively during bioprosthetic AV replacement. Our patient also exhibited AV leaflet fusion, to which both bioprosthetic and native AVs are susceptible during LVAD support. Interestingly, the literature highlights bioprosthetic AV fusion as an inflammatory-mediated process with cellular inflammatory infiltrates at the location of leaflet fusion, whereas this is not observed in native AV fusion.^1,7,13–15^

In cfLVAD patients with bioprosthetic AV replacement, subaortic pannus formation represents a rare complication with distinct clinical implications. Further research is needed to understand this phenomenon and its pathophysiology to create management guidelines aimed at preventing formation and facilitating cfLVAD weaning in affected patients.

## Supplementary Material

ytae592_Supplementary_Data

## Data Availability

No new data were generated or analysed in support of this case report.

## Lead author biography

Dr Rohan Joshua Krishnaswamy is a doctor currently practising at St Vincent’s Hospital, Sydney. His research interests include advanced heart failure and cardiac transplant, durable mechanical circulatory support, and left ventricular assist devices.

## Notes

**Consent:** Written consent for submission and publication of this case report including images and associated text has been obtained from the patient’s next of kin, in line with COPE guidance.

**Funding:** None declared.

## References

[1] Butany J, Leong SW, Rao V, Borger MA, David TE, Cunningham KS, et al Early changes in bioprosthetic heart valves following ventricular assist device implantation. Int J Cardiol 2007;117:e20–e23. 17254647 10.1016/j.ijcard.2006.08.041

[2] John R, Mantz K, Eckman P, Rose A, May-Newman K. Aortic valve pathophysiology during left ventricular assist device support. J Heart Lung Transplant 2010;29:1321–1329. 20674397 10.1016/j.healun.2010.06.006

[3] Doi A, Marasco SF, McGiffin DC. Is a bioprosthetic valve in the aortic position desirable with a continuous flow LVAD? J Card Surg 2015;30:466–468. 25807875 10.1111/jocs.12541

[4] Sakamoto Y, Hashimoto K, Okuyama H, Ishii S, Shingo T, Kagawa H. Prevalence of pannus formation after aortic valve replacement: clinical aspects and surgical management. J Artif Organs 2006;9:199–202. 16998706 10.1007/s10047-006-0334-3

[5] Baradarian S, Dembitsky WP, Jaski B, Abolhoda A, Adamson R, Chillcot S, et al Left ventricular outflow tract obstruction associated with chronic ventricular assist device support. ASAIO J 2002;48:665–667. 12455780 10.1097/00002480-200211000-00016

[6] Rose AG, Connelly JH, Park SJ, Frazier OH, Miller LW, Ormaza S. Total left ventricular outflow tract obstruction due to left ventricular assist device-induced sub-aortic thrombosis in 2 patients with aortic valve bioprosthesis. J Heart Lung Transplant 2003;22:594–599. 12742425 10.1016/s1053-2498(02)01180-4

[7] Feldman CM, Silver MA, Sobieski MA, Slaughter MS. Management of aortic insufficiency with continuous flow left ventricular assist devices: bioprosthetic valve replacement. J Heart Lung Transplant 2006;25:1410–1412. 17178333 10.1016/j.healun.2006.10.004

[8] Patel NH, Guerrero-Miranda C, Hall S, Rafael AE, Brown WM, Bindra AS. Fusion of bovine tissue aortic valve leaflets in a patient with left ventricular assist device. JACC Case Rep 2022;4:604–609. 35615220 10.1016/j.jaccas.2022.02.009 PMC9125526

[9] Kasahara H, Inoue Y, Suzuki S. Recurrent infarctions due to a dome-shaped pannus above the mitral valve prosthesis. J Thorac Dis 2016;8:E130–E132. 26904241 10.3978/j.issn.2072-1439.2016.01.29 PMC4740130

[10] Rose AG, Park SJ. Pathology in patients with ventricular assist devices: a study of 21 autopsies, 24 ventricular apical core biopsies and 24 explanted hearts. Cardiovasc Pathol 2005;14:19–23. 15710287 10.1016/j.carpath.2004.10.002

[11] Teshima H, Hayashida N, Yano H, Nishimi M, Tayama E, Fukunaga S, et al Obstruction of St Jude Medical valves in the aortic position: histology and immunohistochemistry of pannus. J Thorac Cardiovasc Surg 2003;126:401–407. 12928636 10.1016/s0022-5223(03)00702-5

[12] Kostyunin AE, Yuzhalin AE, Rezvova MA, Ovcharenko EA, Glushkova TV, Kutikhin AG. Degeneration of bioprosthetic heart valves: update 2020. J Am Heart Assoc 2020;9:e018506. 32954917 10.1161/JAHA.120.018506 PMC7792365

[13] Mudd JO, Cuda JD, Halushka M, Soderlund KA, Conte JV, Russell SD. Fusion of aortic valve commissures in patients supported by a continuous axial flow left ventricular assist device. J Heart Lung Transplant 2008;27:1269–1274. 19059105 10.1016/j.healun.2008.05.029

[14] Hata H, Fujita T, Ishibashi-Ueda H, Nakatani T, Kobayashi J. Pathological analysis of the aortic valve after long-term left ventricular assist device support. Eur J Cardiothorac Surg 2014;46:193–197. 24335262 10.1093/ejcts/ezt559

[15] Martina JR, Schipper ME, de Jonge N, Ramjankhan F, de Weger RA, Lahpor JR, et al Analysis of aortic valve commissural fusion after support with continuous-flow left ventricular assist device. Interact Cardiovasc Thorac Surg 2013;17:616–624. 23798641 10.1093/icvts/ivt263 PMC3781792

